# Oxidative Stress and Immune System in Vitiligo and Thyroid Diseases

**DOI:** 10.1155/2015/631927

**Published:** 2015-03-09

**Authors:** Roberta Colucci, Federica Dragoni, Silvia Moretti

**Affiliations:** Section of Dermatology, Department of Surgery and Translational Medicine, University of Florence, Ospedale Piero Palagi, Viale Michelangelo 41, 50125 Florence, Italy

## Abstract

Vitiligo is an acquired dermatological disease frequently associated with autoimmune thyroid disorders. Several theories have been proposed so far to unravel the complex vitiligo pathogenesis. Currently, the autocytotoxic and the autoimmune theories are the most accredited hypothesis, since they are sustained by several important clinical and experimental evidences. A growing body of evidences shows that autoimmunity and oxidative stress strictly interact to finally determine melanocyte loss. In this scenario, associated thyroid autoimmunity might play an active and important role in triggering and maintaining the depigmentation process of vitiligo.

## 1. Introduction

Vitiligo is an acquired dermatological disorder characterized by circumscribed depigmented macules due to the loss of functional melanocytes in the epidermis [1]. This pigmentary disease is frequently associated with some autoimmune comorbidities and particularly with autoimmune thyroid disorders (ATD) [2, 3].

Several theories have been proposed so far to disclose vitiligo pathogenesis, such as the autoimmune theory [4], the autocytotoxic theory [5, 6], the neural theory [7], the “impaired epidermal cytokine” theory [8–10], the melanocythorragic hypothesis [11], and the recent inflammatory theory [12], which are currently considered as synergistic in determining the disease [5].

Among the abovementioned theories however, the autocytotoxic and the autoimmune theories are at present the most accredited, since they are sustained by several important clinical and experimental evidences. Current literature reports several evidences suggesting a strict interplay between oxidative stress and immune system, able to trigger and maintain vitiligo depigmentation process and the eventually associated ATD [13].

This review focuses on the most important evidences regarding the role of autoimmunity and oxidative stress, and their interactions, in vitiligo and autoimmune thyroid disorders. Moreover, we suggest a pathogenetic scenario in which the abovementioned autoimmune diseases coexist and sustain each other in a deleterious vicious cycle.

## 2. Vitiligo and Oxidative Stress

In the last decades, a lot of studies suggested that an hypersensitivity to oxidative stress has a crucial role in determining melanocyte degeneration [14]. Vitiligo skin of active phase patients has been demonstrated to display high epidermal levels of reactive oxygen species (ROS), primarily represented by hydrogen peroxide (H_2_O_2_) and peroxynitrite [15, 16]. These alterations are the result of a local and systemic imbalance in enzymatic and nonenzymatic antioxidant systems [17].

Indeed, an abnormal function of the metabolic system of biopterins, leading to high levels of the tetrahydrobiopterin (6BH_4_) and its isomer 7BH_4_, has been demonstrated in vitiligo epidermis [18, 19]. Biopterins act as inhibitors of the enzymes involved in melanogenesis (namely, phenylalanine hydroxylase and tyrosinase) and stimulate the formation of H_2_O_2_ [10–22].

In addition, low levels of enzyme catalase [23–25], an antioxidant enzyme that catalyzes the conversion of hydrogen peroxide in water plus oxygen, and other antioxidant agents such as glutathione peroxidase, glucose-6-phosphate dehydrogenase, superoxide dismutase, and vitamins C and E have been detected both in the epidermis or in the serum of vitiligo patients [15, 25–27], thus suggesting a systemic redox defect in this disease.

An increase of ROS might also be the consequence of an impaired mitochondrial functioning. Indeed, ultrastructural alterations in keratinocytes mitochondria, such as swelling of their membranes and a rearrangement of the cristae, have been demonstrated in the epidermis of vitiligo skin biopsies [28]. Such structural defects directly correlate with a consequent impaired mitochondrial activity, thus leading to an increased generation of reactive oxygen species [28].

As a consequence, local and systemic high levels of H_2_O_2_ are able to alter calcium homeostasis, consequently perturbing the uptake of L-phenylalanine, the amino-acid precursor of tyrosine in melanocytes [29]. In addition, ROS are able to oxidize and inhibit the activity of proopiomelanocortin-derived bioactive peptides ACTH and a-MSH that have crucial role in maintaining efficient melanogenesis, since their release activates a cascade of intracellular signals leading to an upregulation of key enzymes for melanin synthesis, such as tyrosinase and tyrosinase-related proteins TYRP-1 and TYRP-2 (or dopachrome tautomerase, DCT) [30].

ROS accumulation is also able to induce lipid peroxidation, DNA damage, an increased production of proinflammatory and antimelanogenic cytokines, and the loss of functionality of enzymes playing a key role in melanogenesis [31].

Most of the unfavourable effects of H_2_O_2_ accumulation are more frequently observed in keratinocytes and melanocytes derived from perilesional skin, thus suggesting a pivotal role of such area in initiating the depigmentation process.

Moreover, recent studies pointed out the importance of the Nrf2-antioxidant response element (ARE) pathway in regulating vitiligo skin homeostasis under oxidative stress and gave the possibility to explain the hypersensitivity of vitiligo melanocytes to oxidative damage [32–34]. The Nrf2-antioxidant response element (ARE) is indeed a major antioxidant pathway, since it regulates the transcription of stress-related cytoprotective genes, thus protecting cells from oxidative stress and chemical-induced cellular damage. It has been demonstrated that the Nrf2-ARE pathway protects human melanocytes from H_2_O_2_-damage through the induction of downstream antioxidant genes [33], such as heme oxygenase-1 (HO-1). A recent in vitro study showed that vitiligo melanocytes have reduced Nrf2 nuclear translocation and transcriptional activity, which lead to decreased HO-1 expression and aberrant redox balance. Accordingly, authors demonstrated in a clinical setting that serum levels of HO-1 were significantly decreased in vitiligo patients, when compared with healthy controls [34].

Also the Forkhead box class O (FOXO) proteins, a class of transcription factors whose activation leads to the induction of gene codifying for antioxidant proteins, seem to be involved in vitiligo antioxidant impairments [35]. Recently some authors reported a significant association between a specific polymorphism of FOXO3a gene in active vitiligo patients, together with decreased levels of FOXO3a protein, compared to a control group [35].

Finally, a further theory sustaining the pathogenic role of oxidative stress in vitiligo, called the haptenation theory, has been proposed [36]. According to this hypothesis, high levels of hydrogen peroxide (H_2_O_2_) might lead to increased levels of ortho-phenols surrogate substrates of tyrosinase. Authors sustain that vitiligo tyrosinase, due to a genetically controlled polymorphism, could be able to accept the abovementioned substrates, which covalently bind to the enzyme after conversion to reactive ortho-quinone [36]. This process might modify tyrosinase into a neoantigen possibly recognized by the immune system, thus triggering the autoimmune reaction at the basis of the depigmentation process observed in vitiligo [36].

According to all the above reported evidences, in the last decades, some clinical studies showed the beneficial effects of the use of oral and topical antioxidants in association with conventional vitiligo treatment [37–40], thus suggesting the importance to restore the defective antioxidant system in vitiligo patients.

## 3. Vitiligo and Autoimmunity

With regard to the autoimmune theory, it is generally accepted that autoimmunity is strongly implicated in the development of the vitiligo [41], so that this pigmentary disorder is widely considered as autoimmune disease. This theory is sustained by several epidemiological, clinical, and laboratory studies [1, 4, 42, 43]. Elevated organ and non-organ-specific autoantibodies levels have been reported in the serum of vitiligo patients [43]. The frequency of such autoantibodies is variable according to different studies conducted so far [1, 3, 43–46], and their role in vitiligo patients is still quite unknown, mostly if patients positive for such autoantibodies do not display clinical signs of autoimmune associated diseases. However, the finding of elevated organ-specific autoantibodies in vitiligo patients might represent a predictive marker of future overt autoimmune disorders [4].

The involvement of the humoral response in vitiligo is documented by the finding of circulating autoantibodies directed towards melanocytic antigens [47–50], whose levels correlate with disease activity [47]. Such autoantibodies, pertaining to class G immunoglobulins, have been found also in the basal layer of lesional vitiligo epidermis, in association with complement component 3 (C3) deposits [48]. Major melanocytic antigens are the proteins tyrosinase, tyrosinase-related protein-1 (TRP-1), TRP-2, Pmel17 (also called gp100), the transcriptional factors SOX 9 and SOX 10, and the type 1 membrane receptor for melanin-concentrating hormone (MCH-R1) [49–52].

Peripheral blood of patients with vitiligo is also characterized by high frequencies of melanocyte-reactive cytotoxic T cells [39], able to release type B granzyme, perforin, and IFN*γ* [53, 54], while perilesional T-cell infiltration can be found in vitiligo epidermis [54]. It has been demonstrated that perilesional lymphocytic infiltrate is constituted by T cells appearing as skin-homing, polarized toward type-1 effector function, and markedly cytotoxic [54–56].

Moreover, recent findings pointed out a pathogenetic role of TH17 cells in vitiligo [57–59].

Namely, a population of TH17 cells able to release the cytokine IL-17 has been recently found in the epidermis of active vitiligo skin [58, 59]. Accordingly, IL-17 levels have been found increased in the serum and in lesional epidermis of vitiligo patients [57]. This cytokine is able to induce the release of proinflammatory cytokines (namely, IL-1, IL-6, TNF*α*, TGF*β*, GM-CSF, and prostaglandins) from activated immune cells such as fibroblasts, keratinocytes, endothelial cells, and macrophages [59]. This consequent local cytokine network recruits and activates mononuclear lymphocytes or neutrophils, which are strongly implicated in vitiligo pathogenesis. In addition, in vitro studies showed that human cultured melanocytes treated with IL-17A displayed a reduced melanin production, a downregulation of the microphthalmia-associated transcription factor (MITF), which is implicated in the transcription of key genes involved in melanogenesis, and a reduced expression of the m-RNA encoding for the antiapoptotic protein B cell lymphoma gene-2 (BCL2), thus suggesting the unfavourable effects of IL-17A on melanocyte function and survival [59].

Finally, a plethora of novel findings support the crucial role of regulatory T cells (Tregs) in vitiligo pathogenesis [60]. Indeed circulating Tregs, whose function is to maintain peripheral tolerance through the active suppression of self-reactive T-cell activation and proliferation, have been reported to be decreased in vitiligo patients, compared to controls [61, 62]. Accordingly, a remarkable reduction in the number of Tregs has been observed also in the perilesional and lesional skin of vitiligo patients [63]. It is noteworthy that besides a decrease of circulating Treg cells number, patients affected by active vitiligo also display an impaired Tregs function, as demonstrated by their altered capacity to inhibit the proliferation of stimulated CD8^+^T cells and their cytokine production [60]. Possibly, the impaired cytokine network typical of vitiligo might contribute to the reduction and the loss of function of Tregs. Both TGF beta and IL 10 indeed, which are physiological inducers of Tregs function and proliferation, have been found to be decreased in active vitiligo lesions [8, 64, 65], thus leading to an impaired peripheral tolerance. Future strategies for vitiligo treatment will be probably targeted to improve Tregs number and regulatory functions, as shown by recent promising experiments conducted in mice [66].

Taken together, the abovementioned evidences thus suggest the pathogenetic role of both humoral and cell mediated immunity.

Concerning the epidemiological and clinical evidences of an autoimmune theory of vitiligo, the frequent association with organ-specific autoimmune disorders [1, 5, 43] and a positive response to immunosuppressive treatments in vitiligo patients [12, 67] are the most important proofs.

## 4. Interplay between Oxidative Stress and Autoimmunity in Vitiligo

The role played by autoimmunity and oxidative stress in the pathogenesis of vitiligo until now was considered as mutually exclusive. Recent findings instead suggested that these two mechanisms are both involved in the depigmentation process and act in synergism [13]. In autoimmune disorders such as vitiligo, the immune system develops a chronic inflammatory milieu in which ROS accumulate and exert a toxic effect on surrounding cells [13].

Structural or functional melanocytic proteins therefore may be modified by acute and chronic oxidative stress, possibly becoming neoantigens able to trigger autoreactive reactions [68]. Hence, according to this new theory, autoimmunity and oxidative stress interact in initiating and/or amplifying the loss of melanocytes in vitiligo.

A recent paper [69] suggests that oxidative stress and autoimmunity coexist in vitiligo but might play different roles in initiating or perpetrating vitiligo. Namely, in this case control study, anti-melanocyte antibody levels, suggestive of an autoimmune process, and lipid peroxidation levels, which indeed indicate an oxidative stress, were evaluated in both early onset and late duration vitiligo patients. Authors found that lipid peroxidation levels were increased in patients with early onset vitiligo, while, on the contrary, anti-melanocyte antibodies were increased in long duration vitiligo patients [69]. Therefore, oxidative stress rather than autoantibodies might play a major role in initiating vitiligo [69]. Possibly, the consequent accumulation of ROS might secondarily trigger autoimmunity and precipitate the depigmenting process of vitiligo, since ROS might alter the structure of protein involved in melanogenesis, such as tyrosinase, making them more antigenic.

## 5. Pathogenetic Interconnections at the Basis of Vitiligo and Autoimmune Thyroid Comorbidities

Patients with vitiligo have elevated frequencies of associated autoimmune disorders and among them [70, 71] autoimmune thyroid disorders (ATD) are the most frequently found comorbidities [3].

ATD are a group of disease characterized by the presence of autoantibodies directed against thyroglobulin, thyroperoxidase or thyroid-stimulating hormone receptor, which are pivotal thyroid-specific molecules for the production of thyroid hormones. Autoimmune thyroid disorders can be associated or not with thyroid dysfunction. A recent systematic review pointed out that the risk for vitiligo patients to develop ATD diseases is even higher (2.5 fold) compared to nonvitiligo patients, while the risk to develop elevated thyroid antibodies is more than 5-fold higher in vitiligo patients than in nonvitiligo patients [3]. Accordingly, a screening of ATD is recommended in vitiligo patients [3].

A recent study performed by our working group [72] has investigated the presence of an uncommon group of autoantibodies directed against thyroid hormones (triiodothyronine and/or thyroxine) (THAbs), in patients with vitiligo. THAbs represent a class of thyroid autoantibodies showing a very low prevalence in the general population [73, 74] but increased in some thyroid and extra-thyroid autoimmune diseases such as Hashimoto's thyroiditis, Graves' disease, primary Sjögren's syndrome, or rheumatoid arthritis [75]. Even if their pathogenetic role is still quite obscure, a study reported that their presence in nonthyroid autoimmune diseases seem to be predictive of overt ATD [75].

We showed that THAbs have a surprisingly elevated prevalence in vitiligo, higher than in other disease investigated so far, and significantly correlate with active vitiligo, leukotrichia, disease duration, and thyroglobulin antibodies positivity. All together, these results suggest a possible pathogenic role of THAb in vitiligo [72].

As mentioned above, a chronic inflammatory milieu, as can be found in vitiligo, can lead to local and systemic ROS accumulation. To explain our findings, we suggest that ROS increase might be toxic for thyroid, leading the release of large amounts of thyroglobulin proteins that can be more accessible to immune system attack [72].

At the same time, in patients with thyroid autoimmunity increased ROS levels [76] have been demonstrated which might contribute to modify tyrosinase or other melanogenic proteins into neoantigens, leading to the appearance of vitiligo. Thus melanocytic and thyroid system might interact, creating a vicious cycle in which thyroid autoimmune processes give rise to vitiligo lesions, and in turn vitiligo sustains the formation of thyroid autoantibodies, such as THAbs [76].

We suggest that an important role in determining these events might be played also by heavy metals, pollutants, ionizing radiations, and other chemical substances [77] that induce the production of ROS and are considered endocrine disruptors [78]. These agents indeed are able to unfavorably affect thyroid or other endocrine gland functions through a wide range of molecular toxic mechanisms [78]. Therefore, an increased ROS accumulation due to environmental exposure could induce modifications of both melanocytic structures and thyroid proteins, leading to the frequently reported association of vitiligo and thyroid diseases [77].

## 6. Conclusions

Overall, according to the evidences and theories discussed above, we can state that vitiligo has complex pathogenesis in which a pivotal role is played by oxidative stress and immune system. A growing body of evidences indeed shows that autoimmunity and oxidative stress interact and work together in creating a pathway finally able to determine melanocyte loss. In this scenario thyroid autoimmunity, which was so far considered simply as a comorbidity might instead play an active and important role, possibly contributing to trigger and maintain the depigmentation process of vitiligo.
